# Developing a Prototype System for Integrating Pharmacogenomics Findings into Clinical Practice 

**DOI:** 10.3390/jpm2040241

**Published:** 2012-11-20

**Authors:** Casey Lynnette Overby, Peter Tarczy-Hornoch, Ira J. Kalet, Kenneth E. Thummel, Joe W. Smith, Guilherme Del Fiol, David Fenstermacher, Emily Beth Devine

**Affiliations:** 1 Department of Biomedical Informatics and Medical Education, University of Washington, Seattle, WA 98195, USA; 2 Department of Biomedical Informatics, Columbia University, New York, NY 10032, USA; 3 Department of Pediatrics, University of Washington, Seattle, WA 98195, USA; 4 Department of Computer Science and Engineering, University of Washington, Seattle, WA 98195, USA; 5 Department of Radiation Oncology, University of Washington, Seattle, WA 98195, USA; 6 Department of Pharmaceutics, University of Washington, Seattle, WA 98195, USA; 7 Pharmacy Informatics, University of Washington, Seattle, WA 98195, USA; 8 Department of Biomedical Informatics, University of Utah, Salt Lake City, UT 84112, USA; 9 Department of Biomedical Informatics, Moffitt Cancer Center, Tampa, FL 33612, USA; 10 Department of Pharmacy, University of Washington, Seattle, WA 98195, USA; 11 Department of Health Services, University of Washington, Seattle, WA 98195, USA

**Keywords:** electronic health records, clinical decision support systems, pharmacogenomics, personalized medicine, computerized provider order entry, knowledge representation

## Abstract

Findings from pharmacogenomics (PGx) studies have the potential to be applied to individualize drug therapy to improve efficacy and reduce adverse drug events. Researchers have identified factors influencing uptake of genomics in medicine, but little is known about the specific technical barriers to incorporating PGx into existing clinical frameworks. We present the design and development of a prototype PGx clinical decision support (CDS) system that builds on existing clinical infrastructure and incorporates semi-active and active CDS. Informing this work, we updated previous evaluations of PGx knowledge characteristics, and of how the CDS capabilities of three local clinical systems align with data and functional requirements for PGx CDS. We summarize characteristics of PGx knowledge and technical needs for implementing PGx CDS within existing clinical frameworks. PGx decision support rules derived from FDA drug labels primarily involve drug metabolizing genes, vary in maturity, and the majority support the post-analytic phase of genetic testing. Computerized provider order entry capabilities are key functional requirements for PGx CDS and were best supported by one of the three systems we evaluated. We identified two technical needs when building on this system, the need for (1) new or existing standards for data exchange to connect clinical data to PGx knowledge, and (2) a method for implementing semi-active CDS. Our analyses enhance our understanding of principles for designing and implementing CDS for drug therapy individualization and our current understanding of PGx characteristics in a clinical context. Characteristics of PGx knowledge and capabilities of current clinical systems can help govern decisions about CDS implementation, and can help guide decisions made by groups that develop and maintain knowledge resources such that delivery of content for clinical care is supported.

## 1. Introduction

Personalized medicine involves tailoring medical treatment to individual characteristics (e.g., genetic profile). Given the breadth of scope of personalized medicine, drug therapy individualization can provide a more manageable test-bed for studying the informatics issues involved with using electronic health records (EHRs) to support achieving the vision of personalized medicine. We define drug therapy individualization as using genetic or phenotype profile data to predict drug disposition, efficacy, toxicity and clinical outcome. Pharmacogenomics (PGx) is the study of how variations in the human genome affect an individual’s response to medications, and thus provides the evidence-base for making predictions in the context of drug therapy individualization. Clinical decision support (CDS) delivered through use of just-in-time information that combines clinical data with PGx data and knowledge has the potential to improve clinicians’ ability to make personalized drug therapy decisions.

Individualized drug therapy based on genetic testing is often lacking in current formal clinical training for healthcare professionals [1,2]. As our PGx knowledge grows, the traditional approach of expecting clinicians to memorize all pertinent facts for prescribing drugs cannot scale up. In this context, an overarching gap this work aimed to address was the need for education and guidance for health care professionals to support accurately using and interpreting patient specific genetic data for drug therapy individualization in face of ever increasing availability of PGx knowledge and testing. CDS embedded in the EHR might provide a venue for delivering this form of support. Toward this goal, there are CDS technologies that may be adapted to support PGx data and knowledge. In support of this objective, initiatives including those of the US Department of Health and Human Services and the Office of the National Coordinator for Health Information Technology provide infrastructural prerequisites to develop, implement and maintain just-in-time CDS with genetic/genomic knowledge and data in a production system [3]. There are however few studies that explore data and functional requirements for incorporating existing PGx knowledge resources into existing clinical infrastructures to support drug therapy individualization [4,5].

**Figure 1:**
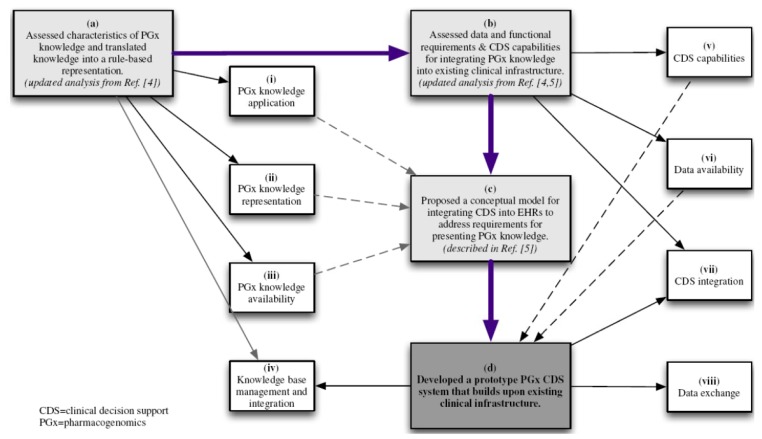
An overview of the rationale behind a prototype pharmacogenomics clinical decision support (CDS) system design. Large boxes indicate methods (**a**–**d**); solid black arrows point to themes of major findings (small boxes **i**–**viii**); dashed arrows point from themes to methods they inform; bold purple lines illustrate the order methods were performed; relationships indicated by grey lines are not described in this manuscript.

Here we provide an overview of our techniques for developing PGx CDS embedded in the EHR as a model for presenting findings from PGx research for drug therapy individualization. As part of this work we refined and extended previously published preliminary assessments of PGx knowledge characteristics [4], data and functional requirements for embedding PGx in the HER [4,5], and CDS capabilities in existing clinical frameworks [5]. Figure 1 illustrates the overall rationale behind the design of the prototype PGx CDS system.

## 2. Methods

The primary focus of this manuscript is to provide an overview of our experiences developing a PGx CDS prototype to support drug therapy individualization (Figure 1d). We used characteristics of PGx knowledge in the US Food and Drug Administration (FDA) drug labels [6] to inform the design of a conceptual model for PGx CDS embedded in the EHR (Figure 1c). Given genomic knowledge is evolving, we refined and extended our previously published methods for translating PGx knowledge into a rule-based representation [4] (Figure 1a). We report the frequency of biomarkers and drugs covered in our updated analysis and the frequencies of rule categories. General categories include: (1) user interface (UI) presentation type (information only, warning, and/or recommendation), (2) analytic phase of genetic testing (pre-analytic, analytic, or post-analytic), and (3) pre-/post- (*i.e*., IF statement/ THEN statement) condition rule pattern (See Section S.1.1 and Section 3.1).

In face of the evolution of PGx we also refined and extended previously published methods for evaluating how local clinical system CDS capabilities align with the data and functional requirements for presenting PGx knowledge derived from existing resources [5] (Figure 1b). A key approach was applying the taxonomy of rule-based decision support content [7] to analyze the functional requirements for PGx CDS and capabilities of UW clinical systems. We report the number of PGx decision support rules that could be implemented given current data availability in local EHRs. Focusing on two domains of practice (oncology and cardiology), we also describe updated functional requirements for PGx CDS, capabilities of UW clinical systems, and how well functional requirements for PGx CDS align with the capabilities of UW clinical systems (See Section S.1.2 and Section 3.2). 

The conceptual model for PGx CDS embedded in the EHR was derived based on findings from evaluations of data and functional requirements (described in Ref. [5]). This manuscript focuses on the development of a prototype system (Figure 1d) informed by this conceptual model (Figure 1c) and findings from updated evaluations of data and functional requirements for integrating PGx knowledge (Figure 1v and Figure 1vi).

### Developing a Prototype Model for Pharmacogenomics Clinical Decision Support

We explored methods for delivering semi-active and active CDS (informed by findings described in Ref. [5]). CDS methods we used to deliver PGx knowledge either currently existed in, or had the potential to be incorporated into, the current UW clinical system infrastructure (See Table 1).

We developed our prototype PGx CDS system within the Cerner® environment, including PowerChart®. Semi-active and active CDS aspects of the conceptual model were implemented in a customized manner using existing tools. Specifically, OpenInfobutton [8,9] for semi-active CDS, and Cerner’s Discern Expert® rules engine [10] for active CDS. CDS was configured in the laboratory review context (e.g., support for interpreting laboratory results) and the medication order entry context (e.g., support for prescribing decisions). The connection between clinical data and PGx knowledge was accomplished with the use of simulated patients and data to “trigger” Discern Expert. In this case, a trigger (event that causes an alert message to display) was ordering a medication for a patient with a particular laboratory result stored (e.g., ordering capecitabine for a patient with a DPYD*2A laboratory result indicating deficient DPD activity). Methods for implementing semi-active CDS and active CDS are described in supplementary materials (Section S.2.2 and Section S.2.3). Preliminary to implementing active CDS we defined simple scenarios specifying triggering conditions (e.g., medication ordered: capecitabine; genomic information: DPYD*2A) (Section S.2.3). We also describe our use of OpenInfobutton and Discern Expert, and limitations we encountered during prototype system implementation (See Section 3.3).

**Table 1:** Clinical decision support methods to deliver PGx knowledge.

CDS delivery method	Description
(CDS implementation type)	
Method 1: PGx link to e-resources	The clinician selects the medication they wish to prescribe and a context-specific link to PGx e-resources appears.
(semi-active CDS)	A context-specific link to PGx e-resources appears next to the genetic test results of interest.
Method 2: Alert message	The clinician enters prescribing information consistent with empirical therapy, clicks the “prescribe” button, and an alert message pops up providing a message relevant to the patients’ genetic test results and the medication being ordered.
(active CDS)	
Method 3: PGx link to e-resources within an alert message	A context-specific link to PGx e-resources appears within an alert message relevant to the patients’ genetic test results and the medication being ordered.
(semi-active CDS that follows active CDS)	

## 3. Results

Findings from updated evaluations of data and functional requirements and CDS capabilities (Figure 1b) informed our decision to incorporate both semi-active and active CDS into a prototype PGx CDS system (See Section 3.1; Figure 1c), and our selection of the local clinical system best suited to implement PGx CDS (See Section 3.2; Figure 1v). Following, we developed a prototype PGx CDS system that builds on existing clinical infrastructure and incorporates semi-active and active CDS (Figure 1d). We summarize limitations to implementing the prototype PGx CDS system; and provide details about our use of the OpenInfobutton and Cerner Discern Expert technologies (See Section 3.3).

### 3.1. Translation of Pharmacogenomics Knowledge into a Rule-Based Representation

Our translation of PGx knowledge into a rule-based representation was updated to include 71 biomarker-drug pairs (Figure 1a). Of these, 26 biomarker-drug pairs were relevant to oncology and cardiology domains of practice, and eleven passed additional exclusion criteria for implementing CDS using Cerner’s Discern Expert and OpenInfobutton (See Section S.2.1). These included five cardiology medications (carvedilol, clopidogrel, metoprolol, propafenone, and warfarin) and six oncology medications (capecitabine, irinotecan, mercaptopurine, nilotinib, tamoxifen, and thioguanine). Across all passages containing PGx knowledge, 565 IF-THEN rules to support PGx clinical decisions were defined. The distribution of biomarkers covered by the IF-THEN rules is shown in Supplementary File 3. Results indicate that 55% of the rules involve genes that encode cytochrome P450 drug metabolizing enzymes (CYP2D6, CYP2C19, CYP2C9). Validated biomarkers that are drug metabolizing genes other than cytochrome P450’s (*i.e*., DPYD, G6PD, NAT, TPMT and UGT1A1) are associated with twelve (or ~17%) of the drugs evaluated in this work. Rules extracted from the drug labels of oncology and cardiology medications account for the majority of all rules defined in this work. In total, there are 239 IF-THEN rules (or ~42% of all rules) defined for 26 oncology and cardiology FDA biomarker-drug pairs. 110 are from the eleven oncology/cardiology medication drug labels that passed additional exclusion criteria.

**Figure 2:**
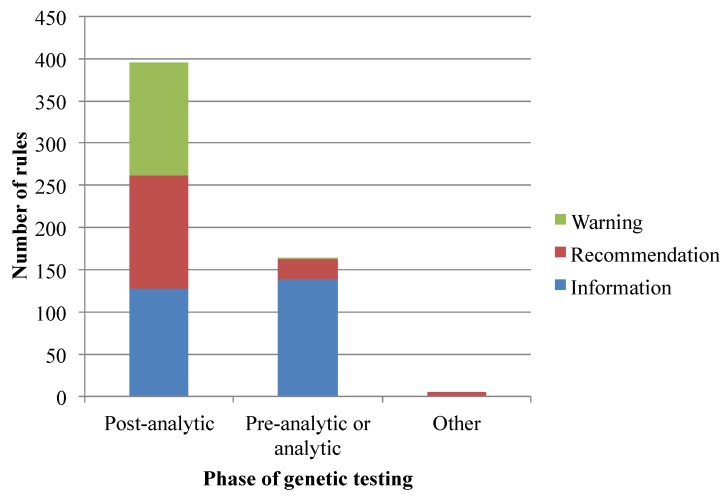
The distribution of 565 IF-THEN rules across phases of genetic testing, stratified by user interface presentation type (warning, recommendation or information only).

In further evaluation, general categories of support provided by each of the 565 IF-THEN rules were determined. Figure 2 summarizes general categories for UI presentation type and support for analytic phases of genetic testing covered by decision support rules derived from drug labels. Overall 47% should be presented as *information only*; 29% should be presented as a *recommendation*; and 24% should be presented as a *warning.* This distribution of UI presentation types illustrates variability in the maturity of PGx knowledge and informed our decision to provide different modes of delivering decision support (*i.e*., semi-active and active CDS). 70% of rules were associated with categories relevant to the post-analytic phase of genetic testing, and 29% were relevant to the pre-analytic phase. The remaining 1% did not clearly fall into any phase of genetic testing. We derived at least one warning rule relevant to the post-analytic phase of genetic testing for 44 of the 71 biomarker-drug pairs. There were fifteen biomarker-drug pairs for which there were warning rules, but no recommendation rules relevant to the post-analytic phase (abacavir – HLA-B*5701, busulfan – Ph Chromosome, carvedilol – CYP2D6, cevimeline – CYP2D6, diazepam – CYP2C19, isoniazid – NAT1;NAT2, metoprolol – CYP2D6, nelfinavir – CYP2C19, quinidine – CYP2D6, rabeprazole – CYP2C19, sodium phenylacetate and sodium benzoate – UCD, tamoxifen – Estrogen receptor, terbinafine – CYP2D6, tramadol and acetaminophen – CYP2D6, and voriconazole – CYP2C19). 

In addition, there were 61 unique pre-/post-condition rule patterns identified. The eight combinations that account for the majority of rule patterns are shown in Supplementary File 4, and accounted for 65% of all decision support rules.

### 3.2. Data and Functional Requirements of Current Clinical Decision Support Systems

The evaluation of clinical data access within UW electronic health records and functional requirements for PGx CDS was updated to assess 565 decision support rules derived from the FDA labeling of 71 biomarker-drug pairs as of May 2011 (See Figure 1b). Altogether, 78% (251/320) of the data elements investigated in this work were captured in UW clinical data repositories. Within each data category, 97% (167/172) of the medications, 57% (39/68) of the conditions, 51% (19/37) of the laboratory values, 59% (23/39) of the demographics, and 75% (3/4) of the procedures were captured. These results are further summarized in Supplementary File 6.

Evaluating CDS requirements for PGx knowledge indicated that the majority of requirements were for computerized provider order entry (CPOE) capabilities. These results are further summarized in Supplementary File 7 (Section S.7.1). Additionally while several of the data element requirements from the updated evaluation remained the same, as expected, the number of rules (or in this case, rule pattern categories) for a given requirement increased substantially. Nineteen out of 61 rule pattern categories were identified in the original evaluation. Also, seven of the top eight IF-THEN rule pattern categories were represented in the initial evaluation. The alignment of these requirements with the current capabilities of UW clinical systems was subsequently determined. To better understand which UW clinical system best supports the requirements for PGx CDS, the percentage of requirements that were and were not supported by each system were investigated and compared. See Supplementary File 7 (Section S.7.2) for support provided by MIND, Cerner, and Amalga. CPOE capabilities in particular were absent from MIND and Amalga, and were supported (but not implemented) in Cerner at the time this evaluation was conducted (Figure 1v). We implemented our PGx CDS prototype in Cerner given that it provided the best support for PGx CDS requirements.

### 3.3. Developing a Prototype Model for Pharmacogenomics Clinical Decision Support

#### 3.3.1. Limitations to Implementing the Pharmacogenomics Clinical Decision Support Prototype

There were two major technical limitations to implementing the PGx CDS prototype: (1) new or existing standards for data exchange in Cerner were required to connect clinical data to PGx knowledge, and (2) Cerner products were not compliant with the *Health Level Seven (HL7) Context-Aware Knowledge Retrieval Standard*, which is adopted by OpenInfobutton for EHR integration. For the purposes of this work, the first limitation was addressed by using simulated patients and clinical data. The second was addressed by modifying how PGx knowledge was represented within Cerner.

Simulated patients with data to trigger the Cerner rules engine were instantiated in the Cerner build environment (a testing environment, separate from the production system environment). Many of the genetic laboratory values of interest to this study exist within the Cerner data repository. However, the laboratory values specific to simple scenarios (See Section S.2.3) defined for eleven medications (See Section 3.1) were unable to be interfaced from the test laboratory system to the decision support rules via Discern Expert at the time this study was completed. Therefore, user-entered dummy laboratory values were defined for each simulated patient and used to trigger alert messages. Other limitations to implementing the prototype system were encountered when representing PGx knowledge in Cerner.

PGx knowledge was represented in Cerner using Discern Expert (for active CDS) and OpenInfobutton (for semi-active CDS). Given that Cerner products could not be configured to access OpenInfobutton, infobuttons were unable to be displayed for the laboratory review context and a workaround was implemented for the medication order entry context. To facilitate implementation of semi-active CDS in the laboratory review context, genetic laboratory values were mocked up with infobutton links in a web-based form external to Cerner. Both active CDS and semi-active CDS were implemented for the medication order entry context. We used Discern Expert to implement active CDS as described in the methods section. Although infobuttons could not be directly configured within Cerner, knowledge resource links generated using OpenInfobutton could be accessed from triggered alert messages via an “EVIDENCE” button (semi-active CDS). Therefore, active CDS followed by semi-active CDS were provided in the medication order entry context.

#### 3.3.2. OpenInfobutton Generated Context-Specific Links

We used OpenInfobutton to generate websites containing links to eight PGx resources overall: CDC Summaries of EGAPP Recommendation Statements [11]; PLoS Currents: Evidence on Genomic Tests [12]; Clinical Pharmacogenetics Implementation Consortium Guidelines [13]; eMedicine: Genomic Medicine Articles [14]; DailyMed [15]; PharmGKB [16,17]; e-PKgene [18]; PubMed [14]. For a subset of the eight resources, we generated multiple links. For example, websites had up to nine links for the medication order entry context, and three for the laboratory review context. Details for two example PGx knowledge resources are described in Supplementary File 5. 

**Figure 3:**
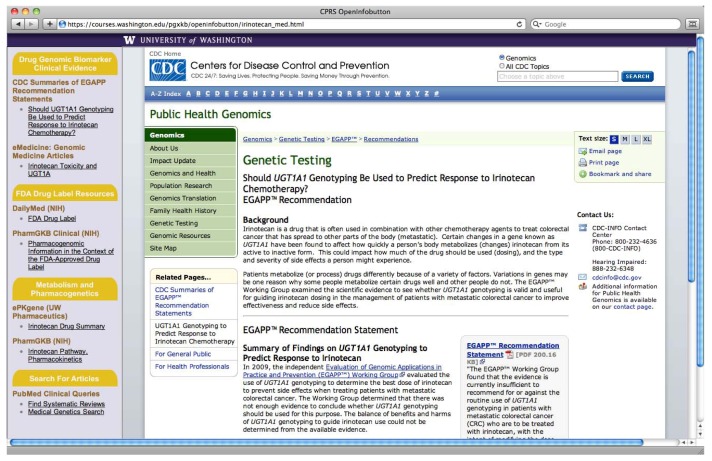
An example website generated using the OpenInfobutton Knoweldge base configured for this project and a customized HTML layout.

Across the full set of resources, only one had an application program interface (API) to support performing a search using a standard medical terminology. Specifically, DailyMed resource uses an RxNorm terminology code specifying a medication name (rxcui) to perform a search. For all other resources medication names and genetic laboratory test results were defined using standard terminologies such as RxNorm and Logical Observation Identifiers Names and Codes (LOINC®) to convey clinical concepts in the EHR to OpenInfobuton. All files were made publically available for download on the OpenInfobutton project webpage [9]. Context-specific websites generated using OpenInfobutton facilitated semi-active CDS in the PGx CDS prototype. Figure 3 illustrates what the HTML-rendered output of OpenInfobutton looked like. The Discern Expert rules engine was used to facilitate active CDS.

The content subsections represented within links generated using OpenInfobutton for nine medications are described in Table 2. The “Drug Genomic Biomarker Clinical Evidence” content subsection contained resources containing guidelines and evidence based synopses, and therefore was considered the subsection that contained the most useful resources. There were no resources categorized under the “Drug Genomic Biomarker Clinical Evidence” content subsection for nilotinib or carvedilol. Alternatively, the majority of the other medications (with high actionable messages) had resources available for the “Drug Genomic Biomarker Clinical Evidence” subsection. 

**Table 2:** Content subsections represented within websites generated using OpenInfobutton for nine medications. “X” indicates all relevant resources, “x” indicates at least one (but not all) relevant resources, and “N/A” indicates no relevant resources were available for a particular medication and content subsection.

Oncology medications	Drug Genomic Biomarker Clinical Evidence	FDA Drug Label resources	Metabolism and Pharmacogenetics	Search for Articles
capecitabine	x	X	X	X
irinotecan	x	X	X	X
nilotinib	N/A	X	N/A	X
mercaptopurine	x	X	X	X
thioguanine	x	X	X	X
**Cardiology medications**				
carvedilol	N/A	X	X	X
clopidogrel	x	X	X	X
propafenone	N/A	X	X	X
warfarin	x	X	X	X

#### 3.3.3. Alert Messages Generated using the Discern Expert Rules Engine

**Figure 4:**
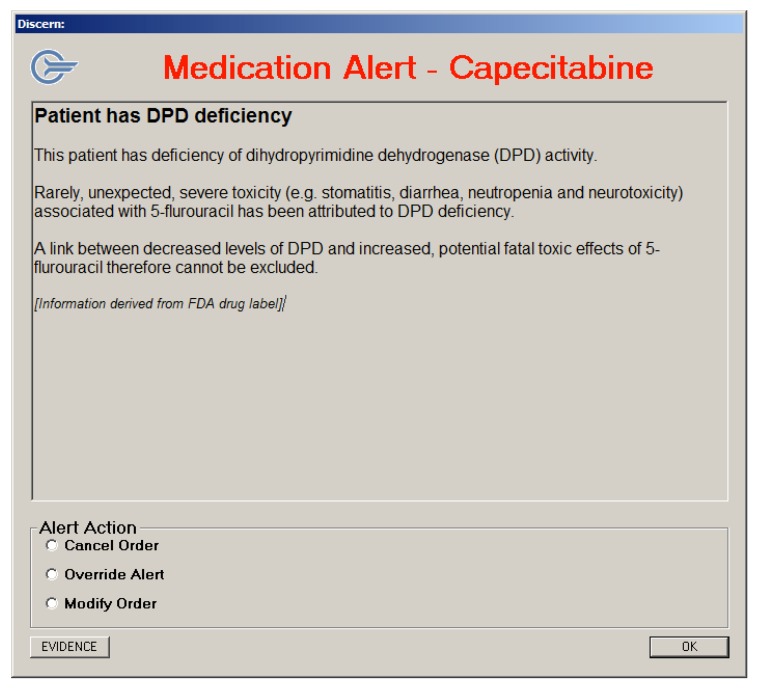
Example Cerner® alert message.

We implemented alert messages for eleven medications (See Section 3.1). Figure 4 illustrates an example alert message that was shown to an ordering physician participating in our study. Offered choices defined within the Action section of the Discern Expert rules included Cancel current order (“Cancel order” alert action in Figure 4), Override rule/keep order (“Override alert” alert action in Figure 4), and Edit current order (“Modify order” alert action in Figure 4). In addition, the alert messages are each configured to include a link to an OpenInfobutton generated website. The websites are accessed via the “EVIDENCE” button shown on the lower left-hand corner of the example alert message in Figure 4.

## 4. Discussion

We presented findings from: (a) an updated translation PGx knowledge into a rule-based representation model; (b) an updated analysis of data and functional requirements, and CDS capabilities; and (c) developing a PGx CDS prototype. This work augments that done by others working towards delivering genomics knowledge to clinicians [19,20,21,22,23,24]. Previous efforts have primarily developed new genomics knowledge bases that are made accessible within local clinical systems. This work, in contrast, first evaluates how local clinical system CDS capabilities align with data, functional and UI presentation requirements for providing just-in-time PGx knowledge derived from existing resources. Then, we developed a PGx CDS prototype given results from the analysis.

Synthesizing findings from this work can shed light on our current understanding of principles for designing and implementing CDS for drug therapy individualization (See Section 4.1). This in turn can inform us about informatics support for genome-based personalized medicine more broadly. In addition, our findings augment well known characteristics of PGx knowledge (e.g., the increasing prevalence of biomarker-drug associations [25] and the evolving maturity of PGx knowledge [26]). We are therefore able to enhance our current understanding of PGx knowledge characteristics in a clinical context (See Section 4.2). 

### 4.1. Implementing Clinical Decision Support for Drug Therapy Individualization

We are able to provide insights into (a) data exchange and data availability in current clinical systems; and (b) CDS functional and UI presentation capabilities to facilitate PGx CDS. 

Considering data exchange and data availability in current clinical systems, our work developing the PGx CDS prototype (Figure 1d) highlighted the need for standards for the exchange of PGx knowledge to facilitate linking genetic laboratory results with knowledge to support their interpretation in a clinical context (Figure 1viii). This limitation might be overcome by incorporating a standardized terminology for genetic laboratory values. For example, the Clinical Bioinformatics Ontology^TM^ (CBO, www.clinbioinformatics.org) developed by Cerner could provide this form of support. The CBO package is freely available, but requires some configuration within Cerner.

We also identified missing UI presentation and functional capabilities needed to properly facilitate PGx CDS integration into UW clinical systems. Our assessment of UI presentation types covered by PGx knowledge (Figure 1a) indicated that maturity of the knowledge varies (Figure 1iii) and may require different modes of delivery (Figure 1c; e.g., semi-active or active CDS). Our assessment of functional requirements (Figure 1b), however, primarily indicated a need for CPOE capabilities (Figure 1vii). In our implementation of the prototype (Figure 1d), while Cerner provided many of the required CPOE capabilities, we found there remained a need for methods for semi-active CDS (Figure 1vii). This finding suggests that our use of the taxonomy of rule-based decision support content [7] to analyze the functional requirements for PGx CDS and capabilities of UW clinical systems may not be sufficient and perhaps should be broadened. Although for this study we were unable to incorporate infobuttons, as standards evolve and vendors begin to adopt them (e.g., HL7 Infobutton standard [27]), incorporation of OpenInfobutton into commercial EHR applications is becoming more feasible. This process will be expedited significantly given the recent inclusion of the Infobutton Standard in the Meaningful Use Stage 2 Standards Certification Criteria for EHR systems [28]. Also, while we found that Cerner products cannot be configured to access OpenInfobutton links, there may be approaches to overcome this limitation that were not explored in this work. For example, within Cerner products, it may be possible to exploit their MPages^TM^ technology to incorporate OpenInfobutton links directly into the PowerChart application. This exploration however, was out of scope for this project. In addition, results from our evaluation of functional capabilities of UW clinical systems may differ from that of other institutions. It is possible that alternative implementations and configurations might result in different functionality. This work would therefore benefit from validation within a healthcare system beyond UW or a decision support engine outside of Cerner. Even so, we evaluated a range of clinical systems in this work, two of which are based on broadly used commercially available clinical data repositories. Findings are therefore likely to be generalizable to other environments.

Overall, it is clear that characteristics of PGx knowledge can help govern decisions about CDS implementation. In addition to providing principles for the design and implementation of CDS from a clinical organization perspective, we can also provide suggestions from the prospective of organizations developing and managing knowledge resources. 

### 4.2. Providing Pharmacogenomics Knowledge within Clinical Information Systems

Considering the perspective of organizations developing and managing knowledge resources, we are able to provide insights into prioritization areas for (a) PGx knowledge development; and (b) PGx knowledge management and knowledge integration.

Our updated assessment of the feasibility of translating PGx knowledge into computable form (Figure 1a) indicated that the majority of all rules derived from FDA drug labeling involve genes that encode cytochrome P450 drug metabolizing genes (Figure 1iii). To further investigate the clinical context in which the decision support rules derived in this work would be most appropriate, rules were each associated with general categories of support (Figure 1a). The majority of rules were associated with categories relevant to the post-analytic phase of genetic testing, and some categories of support were found to be more or less likely to have different designations for UI presentation type (information only, recommendation, and/or warning) (Figure 1iii). Of particular note were indications that there may be more actionable information (*i.e*., recommendations) available in the context of the post-analytic phase of genetic testing when compared to the pre-analytic phase of genetic testing (Figure 1i and Figure 1iii). Although, there were fifteen biomarker-drug pairs of potential high impact for which there were warning rules, but no recommendation rules relevant to the post-analytic phase of genetic testing. These findings suggest a need for actionable PGx knowledge to support interpreting P450s in drug dosing decisions (in the post-analytic phase of genetic testing) (Figure 1i and Figure 1iii), a need for more knowledge to support the pre-analytic phases of genetic testing (Figure 1i and Figure 1iii), and a need for actionable PGx knowledge to support fifteen biomarker-drug pairs of potential high impact (Figure 1i and Figure 1iii). These might be appropriate areas for organizations developing and managing PGx knowledge resources to prioritize. Organizations such as the Clinical Pharmacogenetics Implementation Consortium (CPIC) [13] are already beginning to do this.

Toward understanding prioritization areas for PGx knowledge management and knowledge integration within existing clinical frameworks (Figure 1d), we found that of the PGx knowledge resources we explored, only one had an API to support performing a search using a standard medical terminology (Figure 1iv). Knowledge publishers might better facilitate integration into clinical information systems by the Infobutton Manager by adopting the Context-Aware Knowledge Retrieval Standard (or Infobutton Standard) developed by the HL7 Clinical Decision Support Work Group [27]. The Infobutton Standard specifies the use of standard terminologies in the search engines of resources and makes the configuration of resources easier and the knowledge retrieval process more effective. There is already evidence of an increasing number of knowledge resources becoming HL7 Infobutton-compliant [29].

We also evaluated pre-conditions (IF statement) and post- conditions (THEN statement) of decision support rules (Figure 1a). Our findings showed that the majority of rules were represented within a small number of pre- & post-condition combinations (Figure 1ii). These rule patterns might be useful for prioritizing EHR decision support framework requirements, and for knowledge publishers seeking to represent genomic knowledge such that it supports automatic extraction of relevant data fields. There are also changes being made to drug label content and genomics knowledge at a fast pace, the prevalence of biomarker information in drug labels is increasing, and the maturity of genomic knowledge for clinical use is evolving (Figure 1iii). Given the evolving nature of genomic knowledge, there is a need for modes of identifying updates (that also carry provenance information) in PGx knowledge repositories. There is also a need to capture levels of evidence/certainty of the knowledge. Making these data available would facilitate incorporation of the most relevant and accurate knowledge available in a clinical context. 

## 5. Conclusions

This work enhances our understanding of principles for designing and implementing CDS for drug therapy individualization; and our current understanding of PGx knowledge characteristics in a clinical context. The results highlight several areas that have practical and more general implications for future biomedical and health informatics research. These include the characteristics of PGx knowledge that can help govern decisions about CDS implementation and can help guide decisions made by groups that develop and maintain knowledge resources such that delivery of content in clinical information systems is supported.

This research may be of particular importance for scientific inquiry related to applying our process of evaluating clinical system requirements and capabilities based on knowledge characteristics to deliver personalized healthcare more broadly. This strategy adds to the foundation of CDS system design and has the potential to be applied to systems outside of a local setting.

Another area for scientific inquiry is related to characterizing current PGx knowledge. As part of this work, PGx knowledge was translated into a form capable of being incorporated in current clinical system frameworks. This process highlighted several venues for investigating more automated methods for representing knowledge and new representations of knowledge such that integration into CDS systems is supported.

## Supplementary Files

Supplementary File 1 Developing a Prototype System for Integrating Pharmacogenomics Findings into Clinical Practice (PDF, 590 KB)

## References

[1] Baars M.J., Henneman L., Ten Kate L.P. (2005). Deficiency of knowledge of genetics and genetic tests among general practitioners, gynecologists, and pediatricians: A global problem. Genet. Med..

[2] Menasha J., Schechter C., Willner J. (2000). Genetic testing: A physician’s perspective. Mt. Sinai J. Med..

[3] US Department of Health and Human Services, Office of the National Coordinator for Health Information Technology Personalized Healthcare Detailed Use Case. http://healthit.hhs.gov/portal/server.pt/gateway/PTARGS_0_10731_848111_0_0_18/PHCDetailed.pdf.

[4] Overby C., Tarczy-Hornoch P., Hoath J., Kalet I., Veenstra D. (2010). Feasibility of incorporating genomic knowledge into electronic medical records for pharmacogenomic clinical decision support. BMC Bioinform..

[5] Overby C., Tarczy-Hornoch P., Hoath J., Smith J., Fenstermacher D., Devine E. An evaluation of functional and user interface requirements for pharmacogenomic clinical decision support. Proceedings of 2011 First IEEE International Conference on Health Informatics, Imaging, and Systems Biology (HISB).

[6] US Food and Drug Administration (2011). Table of Pharmacogenomic Biomarkers in Drug Labels. (updated May 2011). http://www.fda.gov/drugs/scienceresearch/researchareas/pharmacogenetics/ucm083378.htm.

[7] Wright A., Goldberg H., Hongsermeier T., Middleton B. (2007). A description and functional taxonomy of rule-based decision support content at a large integrated delivery network. J. Am. Med. Inform. Assoc..

[8] Del Fiol G., Kawamoto K., Cimino J.J. (2011). Open-source, standards-based software to enable decision support. Proc. AMIA Annu. Fall Symp..

[9] Openinfobutton Project Webpage.

[11] US Centers for Disease Control and Prevention CDC Summaries of EGAPP Recommendation Statements (last updated 13 January 2011). http://www.cdc.gov/genomics/gtesting/EGAPP/recommend/.

[12] Gwinn M., Dotson W.D., Khoury M.J. (2010). Plos currents: Evidence on genomic tests—At the crossroads of translation. PLoS Curr..

[13] Relling M.V., Klein T.E. (2011). CPIC: Clinical pharmacogenetics implementation consortium of the pharmacogenomics research network. Clin. Pharmacol. Ther..

[14] Hughes S. (2010). Vanderbilt now also routinely gene testing for clopidogrel metabolizer status. HeartWire.

[15] US National Library of Medicine DailyMed Website..

[16] The Pharmacogenomics Knowledge Base [PharmGKB] Website http://pharmgkb.org.

[17] Hernandez-Boussard T., Whirl-Carrillo M., Hebert J.M., Gong L., Owen R., Gong M., Gor W., Liu F., Truong C., Whaley R. (2008). The pharmacogenetics and pharmacogenomics knowledge base: Accentuating the knowledge. Nucleic Acids Res..

[18] Hachad H., Overby C.L., Argon S., Yeung C.K., Ragueneau-Majlessi I., Levy R.H. (2011). E-pkgene: A knowledge-based research tool for analysing the impact of genetics on drug exposure. Hum. Genomics.

[19] Del Fiol G., Williams M., Maram N., Rocha R., Wood G., Mitchell J. (2006). Integrating genetic information resources with an ehr. Proc. AMIA Annu. Symp..

[20] Kaihoi B., Petersen C., Bolander M. (2005). Providing "just-in-time" medical genomics information for patient care. Proc. AMIA Annu. Symp..

[21] Maviglia S., Yoon C., Bates D., Kuperman G. (2006). Knowledgelink: Impact of context-sensitive information retrieval on clinicians’ information needs. J. Am. Med. Inform. Assoc..

[22] US Department of Health and Human Services Personalized Health Care: Pioneers, Partnerships, Progress. http:www.hhs.gov/myhealthcare/news/phc_2008_report.pdf.

[23] Relling M. Delivery of information: Implementation of pharmacogenomics in clinical practice. Presented at Integrating Large-Scale Genomic Information into Clinical Practice: A Workshop.

[24] Vnencak-Jones C. Pharmacogenetics: From DNA to dosage—Just a click away. Presented at Operationalizing Innovation: Where the Cutting Edge Meets Current Practice (CAP Foundation Confernce Series IV).

[25] Frueh F., Amur S., Mummaneni P., Epstein R., Aubert R., DeLuca T., Verbrugge R., Burckart G., Lesko L. (2008). Pharmacogenomic biomarker information in drug labels approved by the united states food and drug administration: Prevalence of related drug use. Pharmacotherapy.

[26] Deverka P., Doksum T., Carlson R. (2007). Integrating molecular medicine into the us health-care system: Opportunities, barriers, and policy challenges. Clin. Pharmacol. Ther..

[27] Context-aware knowledge retrieval (infobutton) product brief. HL7 International Wiki Site. http://wiki.hl7.org/index.php?title=Product_Infobutton.

[28] (2012). Health Information Technology: Standards, Implementation Specifications, and Certification Criteria for Electronic Health Record Technology, 2014 edition; revisions to the permanent certification program for health information technology. 45 CFR Part 170.

[29] Del Fiol G., Huser V., Strasberg H.R., Maviglia S.M., Curtis C., Cimino J.J. (2012). Implementations of the HL7 context-aware knowledge retrieval ("infobutton") standard: Challenges, strengths, limitations, and uptake. J. Biomed. Inform..

