# Supplementary material for: Developing a Prototype System for Integrating Pharmacogenomics Findings into Clinical Practice
Source: J Pers Med. 2012 Nov 20;2(4):241–56. doi: 10.3390/jpm2040241 (PMC3670105; doi:10.3390/jpm2040241)
Supplement: Supplementary File 1 — Developing a Prototype System for Integrating Pharmacogenomics Findings into Clinical Practice (PDF, 590 KB) [file jpm-02-00241-s001.pdf]

Supplementary Materials

Developing a Prototype System for Integrating Pharmacogenomics Findings into Clinical Practice

Supplementary File 1

S.1.1. Re-evaluating the Translation of Pharmacogenomics Knowledge into a Rule-based Representation

In our original analysis (conducted September 2009), we investigated 28 drug labels of FDA biomarker-drug pairs by: first, identifying passages containing clinically relevant knowledge; and second (whenever feasible) translating passages into a rule-based representation. Given that PGx knowledge contained in drug labels are primarily in free-text form, these steps were performed manually. This evaluation was updated to include drug labels for 71 FDA biomarker-drug pairs listed as of May 2011 [1]. That is, 43 additional biomarker-drug pairs were assessed. In addition, five of the original 28 biomarker-drug pairs were re-assessed completely due to major updates to the labeling (capecitabine – DPD, irinotecan – UGT1A1, nilotinib – UGT1A1, warfarin – CYP2C9 & VKORC1, and clopidogrel – CYP2C19).

In the updated evaluation, passages containing gene specific keywords in DailyMed [2] drug label webpages were extracted in an automated fashion using a Perl script (rather than manually extracted). As previously, passages were manually translated into one or more IF-THEN rules. Given the instantiation of PGx knowledge has inherent uncertainty these rules were approximate. For example, the language used within drug labels varies in certainty (e.g., "patient may require dose reduction" *vs*. "patient requires dose reduction"). Further this instantiation is subject to limitations of the decision support framework chosen (e.g., representing knowledge as an IF-THEN rule *vs*. as an equation). An example of an extracted passage and its translated IF-THEN rules are as follows:

<u>Passage from mercaptopurine FDA drug label (DailyMed HTML page)</u>

- <p>Most patients with heterozygous TPMT deficiency tolerated recommended PURINETHOL doses, but some require dose reduction. Genotypic and phenotypic testing of TPMT status are available. (See CLINICAL PHARMACOLOGY, WARNINGS, and PRECAUTIONS sections.)</p>

<u>Manually translated IF-THEN rules</u>

- IF patient is [being considered for] taking mercaptopurine AND patient is heterozygous TPMT deficient THEN patient likely will tolerate recommended dose, but may require dose reduction.
- IF patient is [being considered for] taking mercaptopurine THEN genotypic and phenotypic testing of TPMT status are available.

In addition, we took three approaches to cluster PGx knowledge contained in FDA drug labels into general categories: (1) user interface (UI) presentation type (information only, warning recommendation), (2) analytic phase of genetic testing (pre-analytic, analytic, or post-analytic), and (3) pre-/post-condition rule pattern. UI presentation types characterize how actionable the "THEN statement" is, of an IF-THEN rule. The methods for assigning UI presentation type are as previously described [3] and

summarized here. A statement is classified: as *information only* if no direct action is specified within the statement, or actions are specified using language with a low degree of certainty (*i.e.*, might, may, could); as a *recommendation* if a clear action is specified using language with a medium to high degree of certainty (*i.e.*, should, will, are, is, must, was, do); and a *warning* if potential consequences are specified (language may be of any degree of certainty). The analytic phases of genetic testing are: the pre-analytic phase that includes events such as determining which genetic test is appropriate to answer a clinical question and collecting/transporting a sample to the test site; the analytic phase that involves sample analysis; and the post-analytic phase that includes reporting and interpretation of results. Pre-/post- condition rule patterns are defined by the pre-condition (the "IF statement") and post-condition ("THEN statement.") For example, the rule "IF patient is [being considered for] taking mercaptopurine THEN genotypic and phenotypic testing of TPMT status are available," has pre-condition: *Drug* and post-condition: *Testing is available*. We report the frequency of biomarkers and drugs covered in our updated analysis and the frequencies of rule categories (See Section 3.1). We also assessed data and functional requirements for each pre-/post-condition rule pattern, and the alignment of these requirements with local clinical system capabilities.

S.1.2. Re-assessing Data and Functional Requirements of Current Clinical Decision Support Systems

The assessment of data and functional requirements for integrating PGx knowledge into existing clinical systems was originally performed in September 2009 with 106 rules derived from the FDA labeling of 28 drugs. The methods and results for this work are described elsewhere. [3] These methods were applied with 565 PGx CDS rules derived from the FDA labeling of 71 biomarker-drug pairs as of May 2011 (derived from S.1.1 above). Data element categories included medications, medical conditions, laboratory values, demographics, and medical procedures. In the original evaluation, 35 data elements categorized as medications, 19 conditions, 20 laboratory values, and one procedure were investigated. In the updated evaluation, 172 medications, 68 conditions, 37 laboratory values, 39 demographics, and four procedures were investigated. Each data element was considered individually, and the number of PGx decision support rules that could be implemented given current data availability in local EHRs was determined (See Section 3.2). Coding systems (*i.e.*, the International Statistical Classification of Diseases and Related Health Problems, Ninth Revision [ICD-9] coding system, the National Drug Code (NDC) coding system, Cerner® Multum Lexicon [Cerner Multum, Inc., Denver CO], and the University of Washington laboratory test guide) helped with distinguishing whether a data element required simple or complex derivation. For example, medication generic and brand names do not require any derivation given that NDC codes exist for the majority of medication names. Simple derivation, however, is required for medication data elements that are chemical classes (e.g., aminosalicylate derivatives) and metabolic classes (e.g., CYP2D6 inducer). Complex derivation is required for data elements for which an IF-THEN rules representation is not sufficient. For example, identifying drugs with a narrow therapeutic index (NTI drugs) ideally requires calculating the dose ratio in dose-response curves or calculating the effect-plasma concentration relationship. This form of calculation would require a more sophisticated representation (e.g., equations).

We next investigated the ability for University of Washington (UW) clinical systems to implement PGx CDS given their current CDS functional capabilities by: (1) analyzing the functional requirements

for PGx CDS; (2) analyzing the functional capabilities of UW clinical systems; and (3) determining how well functional requirements for PGx CDS align with the capabilities of UW clinical systems. Here we provide a brief overview of these methods, although details are described elsewhere [4]. We applied the taxonomy of rule-based decision support content [5] to analyze the functional requirements for PGx CDS and capabilities of UW clinical systems. Specifically, we associated pre-condition (IF statement) and post-condition (THEN statement) rule classifications with taxonomy values (See Section 3.2). Functional capabilities were evaluated by completing informal interviews with individuals who are knowledgeable of UW clinical systems. We assessed (a) the Cerner Millennium® application suite deployed at UW that includes PowerChart® (the inpatient EHR application) and PharmNet® (the inpatient pharmacy application), (b) the UW developed Medical Information Network Database (MIND) system, and (c) the Microsoft™ (now Caradigm) Amalga system. Applications using MIND include MINDscape (a web-based, predominantly view only, EHR application), HealthReach (a patient portal), and U-Link (a referring healthcare provider portal). Applications for Microsoft Amalga include over 300 applications and reports supporting quality improvement, clinical care and operational aspects across UW Medicine. In our initial assessment of how well functional requirements for PGx CDS align with the capabilities of UW clinical systems we focused on two domains of practice (oncology and cardiology) (See Section 3.2). This assessment was updated to include the full set of 565 rules derived from the FDA labeling of 71 drugs (Summer 2011).

Supplementary File 2

S.2.1. Representing Pharmacogenomics Knowledge for Clinical Decision Support

PGx knowledge for CDS was represented for 26 of the 71 FDA biomarker-drug pairs with a particular focus on the oncology domain of practice (17 biomarker-drug pairs) and the cardiology domain of practice (nine biomarker-drug pairs). We narrowed down oncology and cardiology biomarker-drug pairs further to include only commonly used medications (a) with information in their drug labels suggesting that dose modifications may be indicated, and (b) for which the relevant genomic biomarker codes for a drug metabolizing enzyme found in the liver. We also included tamoxifen though it was not an FDA biomarker-drug pair. We therefore represented PGx knowledge for six oncology drugs (capecitabine, irinotecan, mercaptopurine, nilotinib, and thioguanine) and five cardiology drugs (carvedilol, clopidogrel, metoprolol, propafenone, and warfarin) using Cerner's Discern Expert and OpenInfobutton.

S.2.2. Configuring OpenInfobutton to Generate Context-specific Websites

To support semi-active CDS, we enabled context-specific links (a.k.a., "infobuttons") to knowledge resources that can be incorporated into EHR frameworks. These links were enabled by configuring a set of PGx resources within an open source Web service called OpenInfobutton. Taking a particular EHR context as input, OpenInfobutton dynamically generates a set of links to relevant knowledge resources. When a clinician (user context) who is entering medication orders (EHR task) clicks on an infobutton link adjacent to warfarin (concept of interest), these contextual parameters are passed to OpenInfobutton, which returns a list of relevant resources and automated search links in XML (extensible markup language) format.

The major steps taken to utilize OpenInfobuttons in this work were to (a) create a customized stylesheet to render the XML output into the infobutton user interface, and (b) configure the OpenInfobutton Knowledge base. OpenInfobutton's Knowledge Base consists of a database of knowledge resource profiles in XML format. A resource profile describes all the information necessary to enable context-specific links to a resource: (1) the context(s) in which the resource is relevant; (2) the search parameters that are accepted by the resource application program interface (API); and (3) if applicable, the terminologies that are supported by the resource. Profiles were created for each resource that might be included in the websites generated by the Infobutton Manager. The Altova® Authentic® 2011 Desktop Community Edition XML authoring environment was utilized to create profiles for knowledge resources of interest (general process described in Ref. [6]). We used the OpenInfobutton authoring and testing tool [7] to configure a set of knowledge resource profiles for this project (See Section 3.3.2).

Context parameters are defined within resource profiles and are used for *matching* the resource or *searching* against the resource API. All resources considered in this work required configuration of the following context parameters: the *task* (a code representing the task being performed in the EHR e.g., medication order entry), *concept of interest* (the main clinical data of interest in an infobutton request e.g., a medication), and *subtopic* (the specific topic(s) of interest that are associated with the "concept of interest" e.g., a relevant clinical guideline). *Matching* indicates that the context parameter will be

used to determine whether the resource is relevant in a particular EHR context. For example, the DailyMed resource was considered a good "match" only for requests from "medication order entry" infobuttons. *Search* indicates that the resource API is able to process the context parameter for searching content. For example, the rxcui (or RxNorm concept unique identifier) concept of interest was used to search the DailyMed resource.

OpenInfobutton configuration involves specifying an API *Base URL*; *concepts of interest*, and *subtopics*. The Base URL is the URL of the knowledge resource search engine. A *subtopic* is the document subsection or website subsection linked to for a particular concept of interest. Content subsection categories included "Drug Genomic Biomarker Clinical Evidence," "FDA Drug Label resources," "Metabolism and Pharmacogenetics," "Search for Articles," and "Gene Specific Resources." The "Drug Genomic Biomarker Clinical Evidence" content subsection included resources that provide guidelines and evidence based synopses. The "FDA Drug Label resources" content subsection included resources containing FDA information on genomic biomarkers. The "Metabolism and Pharmacogenetics" subsection included resources with evidence of the influence of genetics in the drug elimination process. The "Search for Articles" content subsection included access to systematic review articles and primary literature. The "Gene Specific Resources" content subsection included resources that provide support for interpreting laboratory values in the laboratory review task context. Content subsections were displayed within a customized HTML output that was generated by applying an XSLT stylesheet over the OpenInfobutton XML response. In addition to using OpenInfobutton to facilitate semi-active CDS, Cerner's Discern Expert tool was used to facilitate active CDS in the PGx CDS prototype.

S.2.3. Representing Alert Messages using the Discern Expert Rules Engine

We defined both low and high actionable alert messages to facilitate investigating the impact of implementation method on communication effectiveness and clinical impact. Although that study is beyond the scope of this manuscript, we provide all methods for defining these messages using Cerner's Discern Expert tool. We: (1) identified decision support rule pattern categories associated with each level of actionability, excluding rule patterns that define multiple drugs in the pre-condition (See **Table S1**); (2) defined a simple scenario for each of the eleven medications; (3) for each of the eleven scenarios, identified decision support rules categorized as having low or high actionable rule patterns; (4) for each of the eleven scenarios, defined one low actionable message that combines the post-conditions of the set of rules categorized as having a low actionable rule pattern, and defined one high actionable rule that combines the post-conditions of the set of rules categorized as having a high actionable rule pattern. **Table S2** illustrates simple scenarios, alert messages, and decision support rules derived from drug FDA labels for one of six oncology medications and one of five cardiology medications.

Rules with IF-THEN logic were created with Discern Expert for each alert message (See Section 3.3.3). The logic for all of the rules in this work involves a check that a particular drug is being ordered for a patient with a particular laboratory value. The "THEN" portion of the rule defines what action will take place if the rule logic is satisfied. All of the rules defined in this work involved a Notification action in the form of a text alert message presented in a synchronous manner. The Action of the rule also specifies infobutton links from the alert message to relevant knowledge resources via OpenInfobutton.

Table S1. Decision support rule patterns associated with low and high actionability.

**Pre-condition (IF statement)**	**Post-condition (THEN statement)**	**Actionability**
drug + genotype/phenotype/family_history	toxicity/complications/change_in_pharmacological_ activity	Low
drug + genotype/phenotype/family_history + current_condition/demographic_data/ history_of_condition/ history_of_meds	toxicity/complications/change_in_pharmacological_ activity	Low
drug + genotype/phenotype + current condition/history of condition/history of meds + inpatient/outpatient procedure	toxicity/complications/change_in_pharmacological_ activity	Low
drug + genotype/phenotype/family_history	study_clinical_outcomes	Low
drug + genotype/phenotype/family_history + current_condition/demographic_data/ history_of_condition/history_of_meds	study_clinical_outcomes	Low
drug + genotype/phenotype/family_history + current_condition/demographic_data/ history_of_condition/history_of_meds + inpatient/outpatient_procedure	study_clinical_outcomes	Low
drug + genotype/phenotype/family_history	recommended_treatment_protocol	High
drug + genotype/phenotype/family_history + current_condition/demographic_data/ history_of_condition/ history_of_meds	recommended_treatment_protocol	High
drug + genotype/phenotype/family_history + current_condition/demographic_data/ history_of_condition/history_of_meds + inpatient/outpatient_procedure	recommended_treatment_protocol	High

Table S2. Example alert messages derived from the post-conditions of decision support rules for capecitabine (one of six oncology medications), and carvedilol (one of five cardiology medications).

**Scenario**	**Alert messages**	**Decision support rules**
Medication: Capecitabine<br><br>Genomic Information: DPYD*2A (deficient DPD activity)	Low actionable alert message:<br>**Patient has DPD deficiency**<br><br>This patient has deficiency of dihydropyrimidine dehydrogenase (DPD) activity.<br><br>Rarely, unexpected, severe toxicity (e.g. stomatitis, diarrhea, neutropenia and neurotoxicity) associated with 5-fluorouracil has been attributed to DPD deficiency.<br><br>A link between decreased levels of DPD and increased, potential fatal toxic effects of 5-fluorouracil therefore cannot be excluded.<br><br>High actionable alert message:<br>**Patient has DPD deficiency**<br><br>This patient has known dihydropyrimidine dehydrogenase (DPD) deficiency.<br><br>Capecitabine is contraindicated in this patient.	Low actionable rule(s):<br>• Rule 3.2: IF patient is [being considered for] taking capecitabine AND patient has deficiency of dihydropyrimidine dehydrogenase (DPD) activity THEN rarely, unexpected, severe toxicity (e.g., stomatitis, diarrhea, neutropenia and neurotoxicity) associated with 5-fluorouracil has been attributed to DPD deficiency AND a link between decreased levels of DPD and increased, potential fatal toxic effects of 5-fluorouracil therefore cannot be excluded.<br>High actionable rule(s):<br>• Rule 3.1: IF patient is [being considered for] taking capecitabine AND (patient has known hypersensitivity to capecitabine or to any of its components OR patient has a known hypersensitivity to 5-fluorouracil OR patient has known dihydropyrimidine dehydrogenases (DPD) deficiency OR patient has severe renal impairment) THEN capecitabine is contraindicated in this patient.

Table S2. *Cont.*

Scenario	Alert messages	Decision support rules
Medication: Clopidogrel<br><br>Genomic Information: CYP2C19*2/ *2 (poor metabolizer [8])	Low actionable alert message:<br>**Patient is a CYP2C19 poor metabolizer**<br><br>Copidogrel at recommended doses forms less of that metabolite and has a smaller effect on platelet function in this patient.<br><br>Study results:<br><br>• The majority of published cohort studies show that patients of this status had a higher rate of cardiovascular events (death, myocardial infarction, and stroke) or stent thrombosis compared to extensive metabolizers; and in only one cohort study, the increased event rate was observed only in poor metabolizers.<br>• A crossover study in 40 healthy subjects, 10 each in the four CYP2C19 metabolizer groups, evaluated pharmacokinetic and antiplatelet responses using 300 mg followed by 75 mg per day and 600 mg followed by 150 mg per day, each for a total of 5 days. Decreased active metabolite exposure and diminished inhibition of platelet aggregation were observed in the poor metabolizers as compared to the other groups. When poor metabolizers received the 600 mg/150 mg regimen, active metabolite exposure and antiplatelet response were greater than with the 300 mg/75 mg regimen.<br><br>High actionable alert message:<br><br>**Patient is a CYP2C19 poor metabolizer**<br><br>This patient is a poor metabolizer of CYP2C19.<br><br>Consider alternative treatment or treatment strategies in this patient. An appropriate dose regimen for this patient population has not been established in clinical outcome trials.	Low actionable rule(s):<br>• Rule 5.2: IF patient is [being considered for] taking clopidogrel AND patient is a CYP2C19 poor metabolizer THEN clopidogrel at recommended doses forms less of that metabolite and has a smaller effect on platelet function in patient.<br>• Rule 5.16: IF patient [is being considered] for clopidogrel AND patient is a poor metabolizer of CYP2C19 THEN A crossover study in 40 healthy subjects, 10 each in the four CYP2C19 metabolizer groups, evaluated pharmacokinetic and antiplatelet responses using 300 mg followed by 75 mg per day and 600 mg followed by 150 mg per day, each for a total of 5 days. Decreased active metabolite exposure and diminished inhibition of platelet aggregation were observed in the poor metabolizers as compared to the other groups. When poor metabolizers received the 600 mg/150 mg regimen, active metabolite exposure and antiplatelet response were greater than with the 300 mg/75 mg regimen<br>• Rule 5.18: IF patient is [being considered for] taking clopidogrel AND (patient is an intermediate metabolizer of CYP2C19 OR patient is a poor metabolizer of CYP2C19) THEN the majority of published cohort studies show that patients of this status had a higher rate of cardiovascular events (death, myocardial infarction, and stroke) or stent thrombosis compared to extensive metabolizers; and in only one cohort study, the increased event rate was observed only in poor metabolizers.<br>High actionable rule(s):<br>• Rule 5.5: IF patient is [being considered for] taking clopidogrel AND patient is identified as a CYP2C19 poor metabolizer THEN consider alternative treatment or treatment strategies in the patient.<br>• Rule 5.17: IF patient [is being considered] for clopidogrel AND patient is a poor metabolizer of CYP2C19 THEN an appropriate dose regimen for this patient population has not been established in clinical outcome trials.

Supplementary File 3

Figure S1. Distribution of biomarkers covered by 565 decision support rules extracted from 71 FDA drug label.

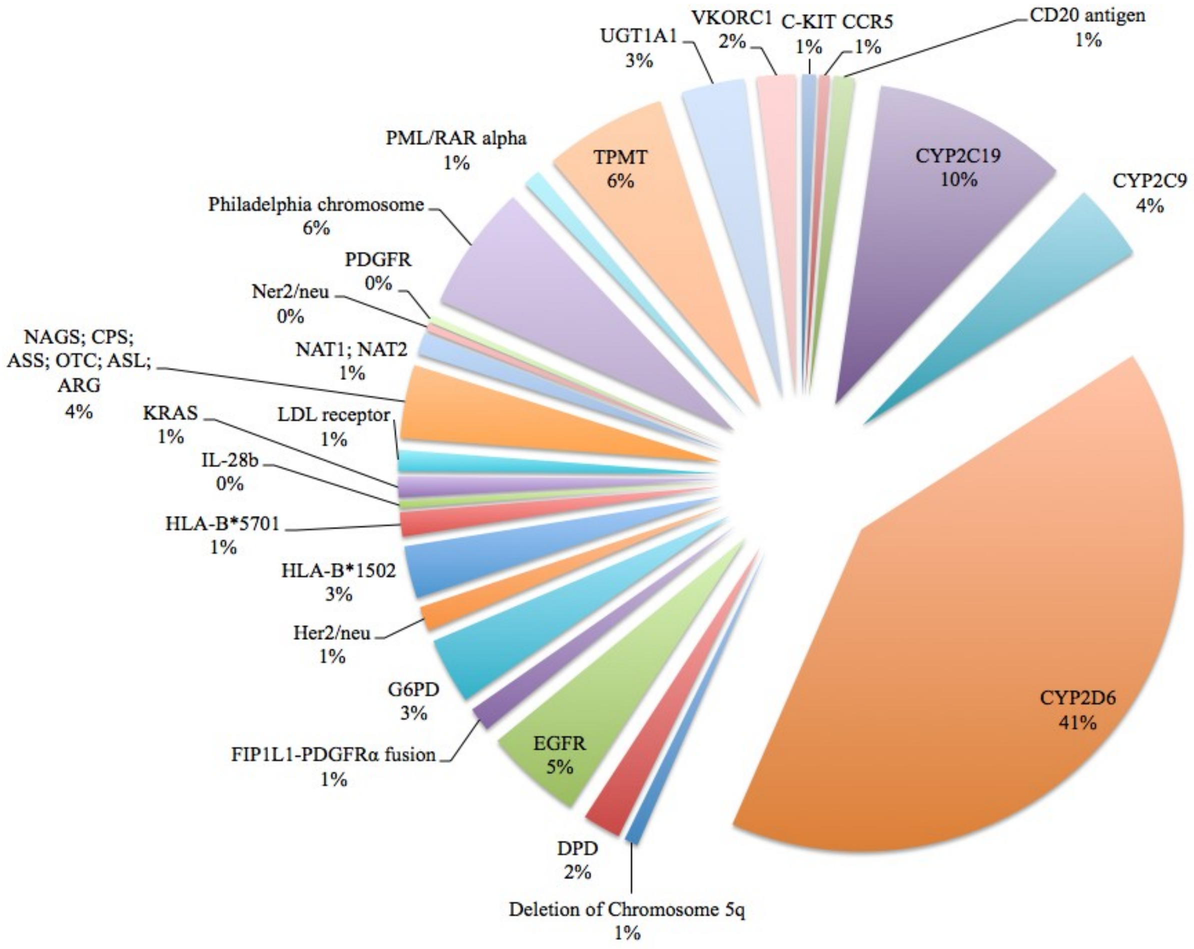

Supplementary File 4

Table S2. Top eight IF-THEN rule patterns seen for 565 decision support rules extracted from 71 FDA drug labels. Each row includes the pre-condition (column 1), post-condition (column 2) and an example rule designated as having the pre- & post- condition combination (column 3).

IF statement	THEN statement	Example decision support rule
drug	pharmacological_activity_ with_involvement_of_gene/ protein	IF patient is [being considered for] taking Esomeprazole THEN Esomeprazole is extensively metabolized in the liver by CYP2C19 and CYP3A4
drug + genotype/phenotype/ family_history	recommended_treatment_ protocol	IF the patient is taking Celecoxib AND the patient has a CYP2C9 variant AND the variant causes poor metabolizm THEN Celecoxib should be administered to the patient with caution
drug + genotype/phenotype/ family_history	toxicity/complications/ change_in_pharmacological _activity	IF patient has an inherited deficiency of the enzyme thiopurine methyltransferase (TPMT) AND patient is [being considered for] taking thioguanine THEN patient may be unusually sensitive to the myelosuppressive effects of thioguanine, and may be prone to developing rapid bone marrow suppression following initiation of thioguanine therapy
drug + genotype/phenotype/ family_history + current_condition/demograph ic_data/history_of_condition/ history_of_meds	recommended_treatment_ protocol	IF patient is [being considered for] taking ACZONE AND (patient is glucose 6-phosphate dehydrogenase deficient OR patient has a history of anemia) THEN patient is at risk; and routine follow-up for complete blood count and reticulocyte count should be implemented.
drug + population	probability/frequency_of_ having_variants_in_ population	IF the patient is taking Carbamazepine AND patient is Asian AND patient is from Taiwan THEN About 10% of the population is reported positive (for HLA-B*1502) in Taiwan
drug1 + drug2/ current_med_list	recommended_treatment_ protocol	IF patient is [currently] taking NUEDEXTA AND (patient is [being considered for] taking medications that are primarily metabolized by CYP2D6 AND medications have a relatively narrow therapeutic index) THEN medications should be initiated at a low dose.
drug1 + drug2/ current_med_list	toxicity/complications/ change_in_pharmacological _activity	IF patient is [being considered for] taking clozapine AND patient is taking certain drugs that are metabolized by P450 2D6 including antidepressants THEN drugs metaboized by P450 2D6 may inhibit the activity of P450 2D6 and thus may make normal metabolizers resemble poor metabolizers with regard to concomitant therapy with other drugs metabolized by this enzyme system, leading to drug interaction.
genotype/phenotype/family_ history + current_condition/ demographic_data/history_of _condition/history_of_meds	recommended_treatment_ protocol	IF the patient is an adult AND the patient is infected with an HIV virus that is CCR5-tropic AND the virus is resistant to multiple antiretrovirals AND the patient has evidence of viral replication, THEN treat the patient with SELZENTRY

Supplementary File 5

Table S3 provides details about a representative PGx knowledge resource configured for the "medication order entry" task context. OpenInfobutton knowledge resources configured for this task are made available via a prototype implementation of the CDS Method 3 described in **Table 1**. Specifically, the electronic resources made available in an OpenInfobutton response from an alert message include a subset or all of the resources configured for the medication order entry task. **Table S4** provides details about an example PGx knowledge resource configured for the "laboratory review" task context, and that are also made available within a prototype implementation of the CDS Method 1 described in **Table 1**. Specifically, the electronic resources made available within an OpenInfobutton response from genetic laboratory results includes a subset or all of those configured for the laboratory review task. Knowledge resources provided in both task contexts are made available to physicians to support their interpretation of patient genetic laboratory values when prescribing a medication.

Table S3. Example pharmacogenomics knowledge resource included in OpenInfobutton generated websites and configured for the Medication Order Entry context (This is one of nine such examples).

Pharmacogenomics Knowledge Resource Details (Medication order entry context)	Content subsection
Resource: **CDC Summaries of EGAPP Recommendation Statements [9]**<br>Base URL: **http://www.cdc.gov/genomics/gtesting/EGAPP/recommend/**<br>Concept of interest: **Irinotecan**<br>Subtopic: **Should UGT1A1 Genotyping Be Used to Predict Response to Irinotecan Chemotherapy? EGAPP Recommendation**	Drug Genomic Biomarker Clinical Evidence

Table S4. Example pharmacogenomics knowledge resource included in OpenInfobutton generated websites and configured for the Laboratory Review context (This is one of three such examples).

Pharmacogenomics Knowledge Resource Details (Laboratory review context)	Category of evidence
Resource: **ePKgene [10]**<br>Base URL: **https://courses.washington.edu/pgxkb/images/** ***(NOTE: URL was active only for this study)***<br>Concept of interest: **CYP2C19**<br>Subtopic: **CYP2C19 Gene Summary**<br>Concept of interest: **CYP2C9**<br>Subtopic: **CYP2C9 Gene Summary**<br>Concept of interest: **CYP2D6**<br>Subtopic: **CYP2D6 Gene Summary**<br>Concept of interest: **UGT1A1**<br>Subtopic: **UGT1A1 Gene Summary**	Gene Specific Resources

Information about the API and about the OpenInfobutton configuration is described for example resources in column 1 of **Table S3** and **Table S4**. Specifically, the API *Base URL*; the configured *concepts of interest*, and the configured *subtopics* are described.

Supplementary File 6

Figure S2. Availability of data elements by category. Data element categories are listed on the x-axis. The last category, "All categories" indicates values across all data element categories. The y-axis is the number of data elements that are: captured (no derivation required), captured (simple derivation required), not captured (complex derivation required), and not captured (unable to be derived) for each category.

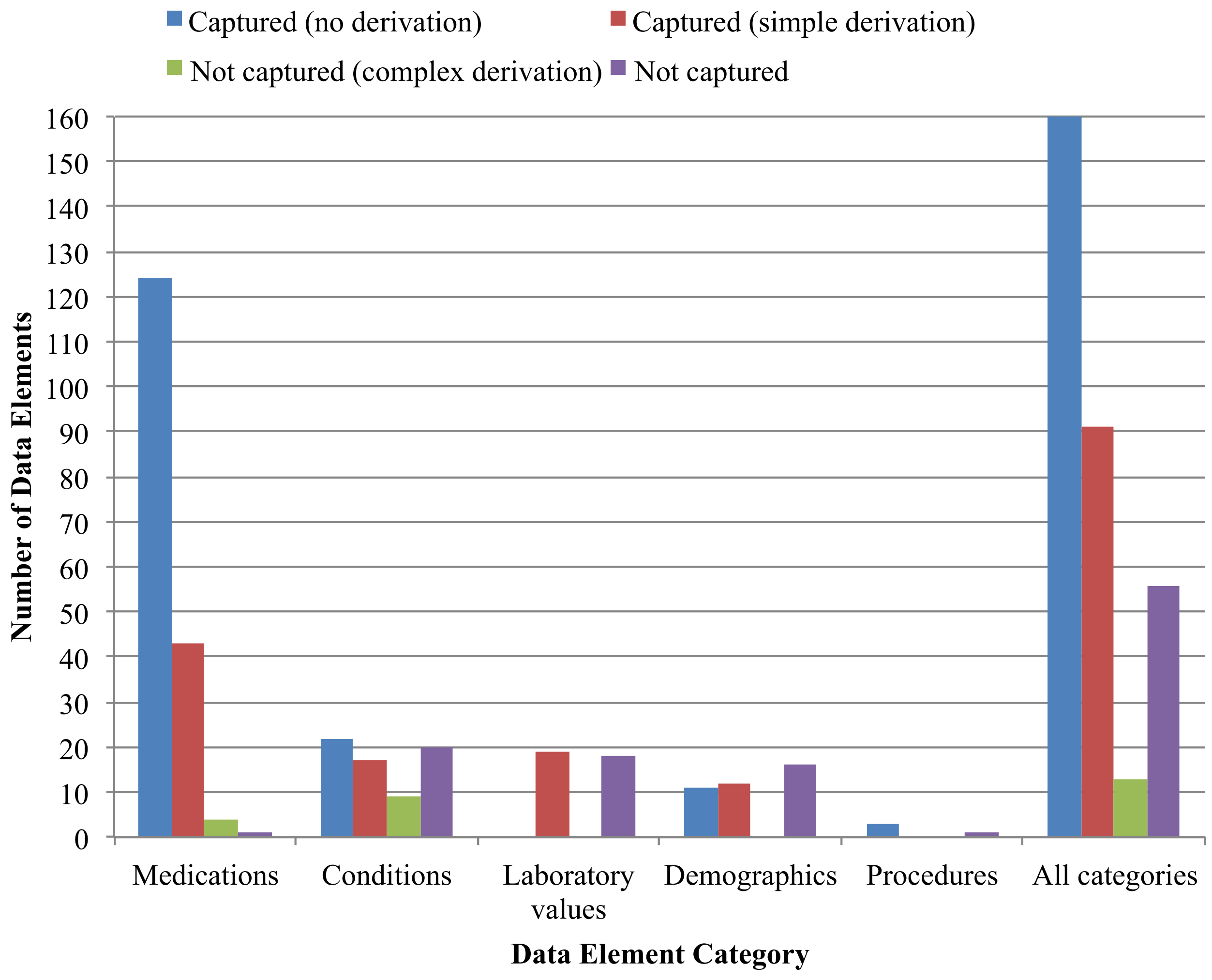

Supplementary File 7

Clinical decision support (CDS) capabilities in existing clinical frameworks were previously reported in Ref [4]. Our initial evaluation of the functional requirements for PGx CDS was performed with 110 decision support rules derived from the drug labels of ten medications (five oncology and five cardiology medications). [4] The updated evaluation, performed Summer 2011, investigated the CDS functional requirements for all 565 decision support rules derived from the FDA labeling of 71 biomarker-drug pairs.

S.7.1. Functional Requirements for Pharmacogenomics Clinical Decision Support

Similar to the previous findings, 6/9 of the triggers, 4/7 of the interventions and 9/14 of the offered choices were required across all decision support rules. However, the discrete data element requirements increased from 6/18 (when 110 rules were evaluated) to 13/18 discrete data elements required (when 556 rules were evaluated). The majority of the functional requirements were for computerized provider order entry (CPOE) capabilities. 90% (55/61) rule patterns required an "order entered" trigger; 100% required a "notify" intervention; and 56%, 61%, and 59% required "override rule/keep order," "cancel current order," and "edit current order," offered choices (respectively).

S.7.2. Pharmacogenomics Clinical Decision Support Requirements Supported by of UW Clinical Systems

Figure S3. Percentage of pharmacogenomics clinical decision support requirements supported and not supported by UW MIND system, Cerner Millenium and Microsoft Amalga (by functional requirement).

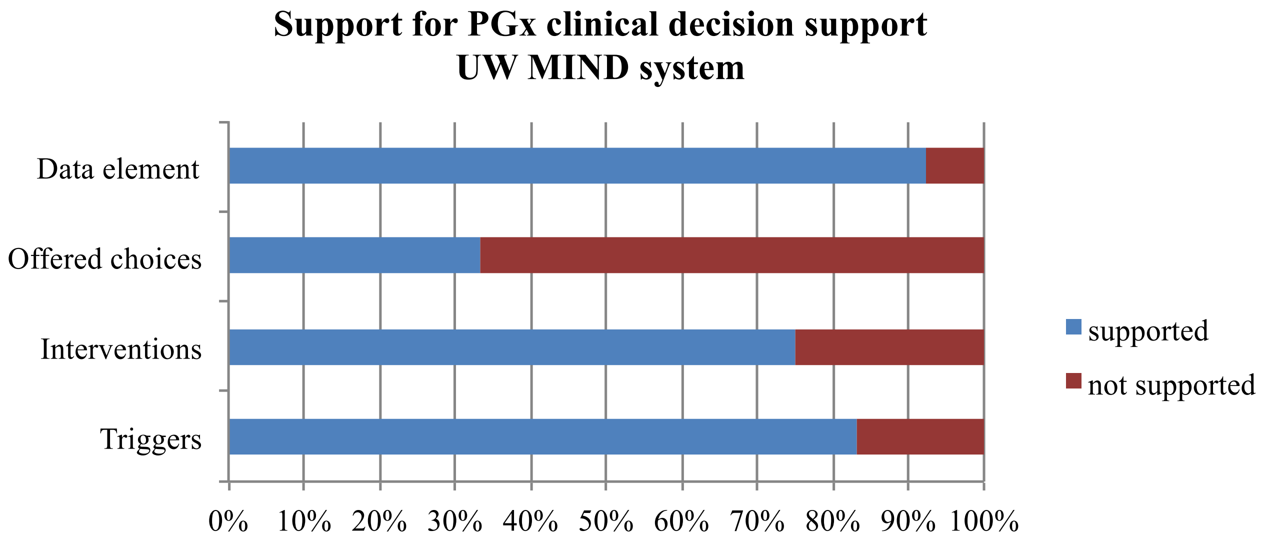

Figure S3. *Cont.*

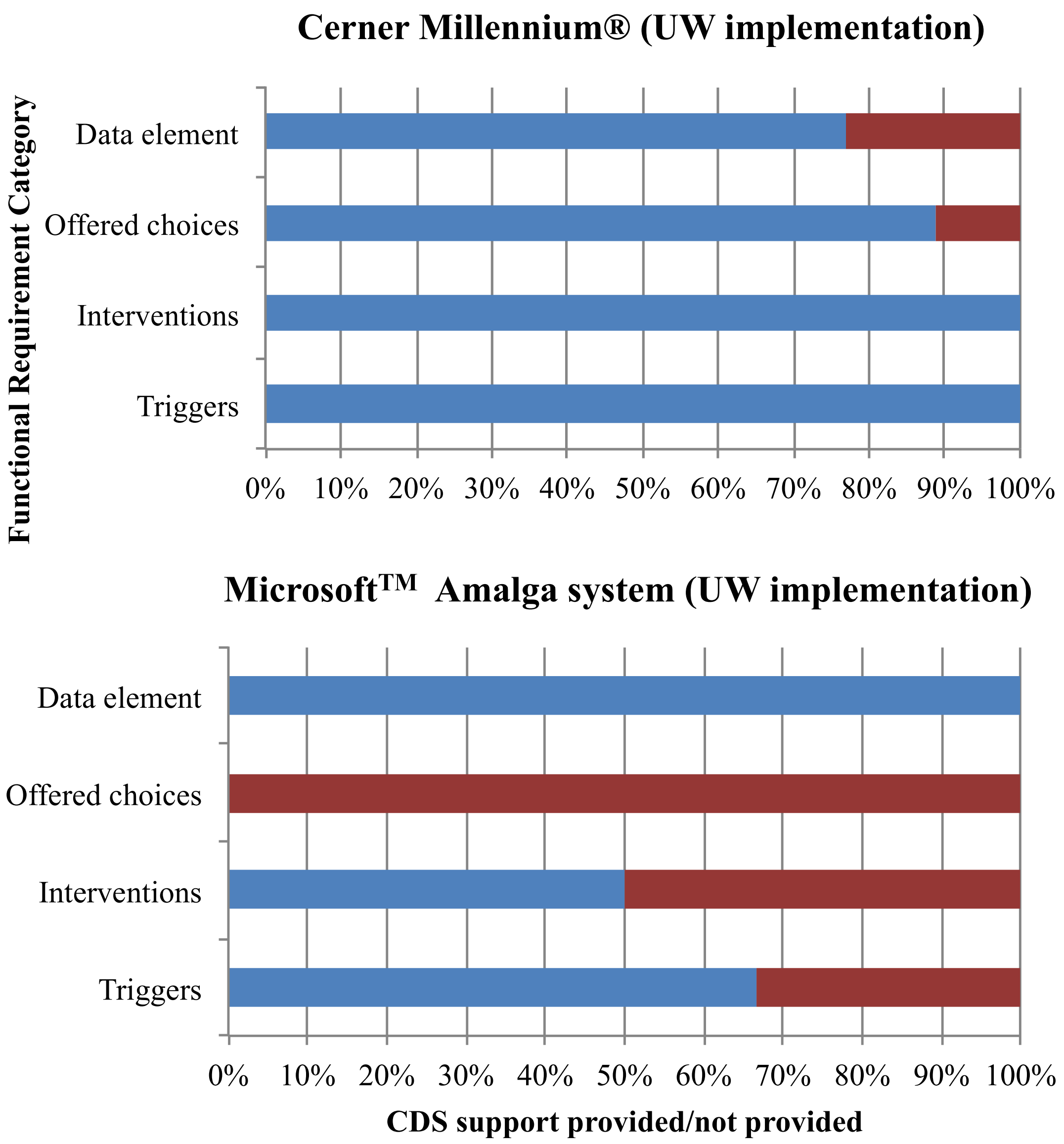

References and Notes

1. US Food and Drug Administration. Table of valid genomic biomarkers in the context of approved drug labels (updated may 2011). Available online: http://www.fda.gov/drugs/scienceresearch/researchareas/pharmacogenetics/ucm083378.htm (accessed on 11 October 2012).
2. US National Library of Medicine, DailyMed web site. Available online: http://dailymed.nlm.nih.gov/dailymed/about.cfm (accessed on 11 October 2012).
3. Overby, C.; Tarczy-Hornoch, P.; Hoath, J.; Kalet, I.; Veenstra, D. Feasibility of incorporating genomic knowledge into electronic medical records for pharmacogenomic clinical decision support. *BMC Bioinform.* **2010**, *11* (Suppl. 9), S10.
4. Overby, C.; Tarczy-Hornoch, P.; Hoath, J.; Smith, J.; Fenstermacher, D.; Devine, E. An evaluation of functional and user interface requirements for pharmacogenomic clinical decision support. In Proceedings of 2011 First IEEE International Conference on Health Informatics, Imaging, and Systems Biology (HISB), San Jose, CA, USA, 26–29 July, 2011.

5. Wright, A.; Goldberg, H.; Hongsermeier, T.; Middleton, B. A description and functional taxonomy of rule-based decision support content at a large integrated delivery network. *J. Am. Med. Inform. Assoc.* **2007**, *14*, 489–496.
6. Del Fiol, G. OpenInfoButton Tailoring Guide. Available online: http://www.openinfobutton.org/file-cabinet/OpenInfobuttonTailoringguide.docx (accessed on 11 October 2012).
7. OpenInfobutton Project Testing Tool. Available online: http://webapps.chpc.utah.edu:8080/infobutton-service/InfobuttonQA.html (accessed on 11 October 2012).
8. University of Washington Department of Pharmaceutics. ePKgene, Cyp2c19 gene polymorphism summary (last updated March 2011). Available online: http://www.pharmacogeneticsinfo.org (accessed on 2 March 2011).
9. US Centers for Disease Control and Prevention, CDC Summaries of EGAPP Recommendation Statements (last updated 13 January 2011). Available online: http://www.cdc.gov/genomics/gtesting/EGAPP/recommend/ (accessed on 11 October 2012).
10. University of Washington Department of Pharmaceutics, ePKgene, Impact of Genetics on Drug Exposure. Available online: http://www.pharmacogeneticsinfo.org (accessed on 11 October 2012).
